# Position-Sensitive Bulk and Surface Element Analysis of Decorated Porcelain Artifacts

**DOI:** 10.3390/ma15155106

**Published:** 2022-07-22

**Authors:** László Szentmiklósi, Boglárka Maróti, Szabolcs Csákvári, Thomas Calligaro

**Affiliations:** 1 Nuclear Analysis and Radiography Department, Centre for Energy Research, 1121 Budapest, Hungary; maroti.boglarka@ek-cer.hu (B.M.); csaszabolcs@gmail.com (S.C.); 2 Centre de Recherche et de Restauration des Musées de France, Palais du Louvre, 75001 Paris, France; thomas.calligaro@culture.gouv.fr

**Keywords:** X-ray Fluorescence Spectrometry, Prompt-gamma activation analysis, porcelain, non-destructive composition

## Abstract

Non-destructive characterization of decorated porcelain artifacts requires the joint use of surface-analytical methods for the decorative surface pattern and methods of high penetration depth for bulk-representative chemical composition. In this research, we used position-sensitive X-ray Fluorescence Spectrometry (XRF) and Prompt-gamma activation analysis (PGAA) for these purposes, assisted by 3D structured-light optical scanning and dual-energy X-ray radiography. The proper combination of the near-surface and bulk element composition data can shed light on raw material use and manufacturing technology of ceramics.

## 1. Introduction

The ancient Greek term “keramos” means clay; the derived phrase “ceramics” nowadays refers to all clay-based materials that have undergone an irreversible physical-chemical transformation during firing. Porcelain is a special class of ceramics that is of fine-grained body, usually translucent to visible light, and stands out for its whiteness. The challenge in the elemental composition analysis is common to all ceramic types due to their similar compositions.

Porcelain has a rich history of over a thousand years. The first porcelain objects were created in China [1,2] and distributed via trading routes to India, the Middle East [3,4,5], and later to Europe. Local production of porcelain appeared in Europe in the 16th century and became widespread in the 18th century, with the establishment of traditional manufacturing workshops [3,4]. Consequently, porcelain artifacts are abundant and in well-preserved conditions but show significant differences in terms of composition, structure, and visual appearance.

Raw materials, the mixing proportions, the formation technology, the glaze, and the firing conditions all influence the bulk material properties. Although the structure changes during the firing step of preparation and the raw material dehydrates, the characteristic bulk element composition patterns from the raw materials are preserved. Typically, it consists of 65–80 m% SiO_2_, 8–22 m% Al_2_O_3_, and the remaining 0.5–3 m% is of various oxides (e.g., Na_2_O, K_2_O, MgO, CaO, Fe_2_O_3_, ZrO) [6]. The color palette used for decoration also varies between workshops, but the recipes remained stable over the years. Therefore, both bulk and near-surface composition offer discrimination and may be used to answer questions related to authenticity [7], classification, or manufacturing.

Most studies on porcelain artifacts are being made with surface-confined analytical techniques, such as portable XRF [8,9], Raman [10], or PIXE/PIGE. [11] In this paper, we focus on contrasting the elemental compositions at the surface and in bulk and on the methodology to collect such information. There is no single non-destructive method with penetration depth tunable in the required thickness range and adequate sensitivity; this calls for a combination of non-destructive element analysis techniques [12,13]. Here, we made use the different information depths of X-rays and neutrons/gamma rays: the concentration difference of certain elements between the surface-confined results and the bulk indicates its presence at the surface decoration, or vice versa, in the base material.

## 2. Materials and Methods

Several routine instrumental element-analytical techniques [14] either require destructive sample preparation, such as powdering, homogenization, and dissolution, or they are microdestructive and leave visible traces on the object’s surface after the analysis. These methods are out of scope when studying valuable heritage objects. The analytical technique based on the neutron-induced capture gamma-rays, i.e., Prompt-gamma activation analysis (PGAA) [15], and the (portable) X-ray Fluorescence Spectroscopy (XRF) [16], are both contactless and non-invasive measurement techniques for the direct, bulk- or surface-representative element analysis of solid samples, respectively. Neutrons and energetic gamma rays, unlike X-ray photons, have penetration depths as high as a few cm; this makes them appropriate for measuring the bulk composition of solid samples [17].

Our goal with this study is to benchmark the well-established X-ray Fluorescence Spectrometry (XRF) and Prompt-gamma activation analysis (PGAA) in position-sensitive applications, where both lateral and in-depth variation of the elemental concentrations are expected. Most of the PGAA data [18,19] reported in the literature are bulk concentrations, and few attempts were made for any spatial or surface-bulk discrimination. Further, the 2D XRF scanning methodology [20,21] is mostly applied to flat shreds [22,23,24], while in this work, we attempted to extend XRF scanning to non-flat objects by driving the positioner with a digital mesh geometry obtained by 3D optical scanning.

### 2.1. Benchmark Object

To benchmark analytical techniques applicable to decorated porcelain characterization, a traditional porcelain pot object (*boîte mézy*) was handcrafted at the Manufacture Nationale de Sèvres, France, using traditional techniques and raw materials (paste, glaze, flux, and pigments). The body, which has a diameter of 115 mm, was shaped from a traditional paste (70 m% kaolin) on the potter’s wheel and fired. The glaze was applied by dipping, and it was again fired. It was subsequently decorated with complex patterns of high chemical contrast (Au, Co, …), fired, and burnished. These fine details were to challenge the discrimination capabilities of the techniques. It featured a painted Colibri bird and a flower, typical motives of the 18^th^ century, a gold inlay at the center, while a quarter of its lid was covered with traditional blue paint, *Bleu de Sèvres*. In addition, it had the reference color palette painted, allowing the identification of the pigments. The pigment palette corresponded to those used in the 19th and 20th centuries [25]: Pb-Si-B flux with colorants Cr, Co, Fe, Mn, Au, and Cu. The decorations of this object have been extensively characterized by position-sensitive PIXE at the AGLAE facility [26].

### 2.2. Geometry-Digitalization via 3D Structured-Light Scanning or Neutron Tomography

**Figure 1:**
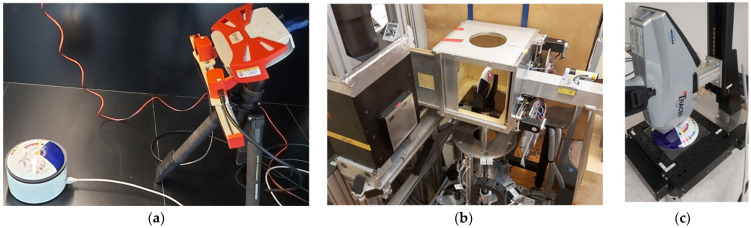
(**a**) The 3D structured-light scanning procedure, (**b**) the experimental setup of the PGAI measurement at the NIPS-NORMA facility of the BNC, and (**c**) position-sensitive xyz XRF scanning device.

Preparatory to the element analysis, fragile or irregular-shaped objects were frequently 3D scanned with a RangeVision SMART [27] structured-light optical scanner (Figure 1a), or their geometries were determined using the advanced surface determination feature of the VG Studio MAX 3.2 software [28] from volumetric X-ray or neutron tomograms, to obtain 3D digital surface mesh of the objects. In addition to just displaying the visual features, we made use of these digital models during position-sensitive element analysis experiments, as discussed hereafter.

### 2.3. Visualization of the Decoration by Dual-Energy X-ray Imaging

In medical imaging, dual-energy X-ray imaging [29] is a well-established practice. Here, we applied this approach by taking radiograms at a low-energy (35 keV) and a high-energy (200 keV) voltage setting of the X-ray generator tube to enhance sensitivity for the surface and bulk, respectively. For each setting, outlier removal, beam and dark image corrections were made, and the logarithm of the images was taken, resulting in the product of the linear attenuation coefficients (μ) and the material thickness (d) in a pixel at an (x,y) coordinate. The quantity (μ × d) differs considerably for the different energy beams, providing enhanced contrast to the otherwise very thin paint layer.

### 2.4. Position-Sensitive Prompt-Gamma Activation Analysis (PGAI) for Bulk Characterization

Prompt-gamma activation analysis (PGAA) is a potent in situ and contactless elemental analysis technique based on the radiative neutron capture nuclear reaction [30]. During irradiation with a well-collimated beam of slow neutrons, characteristic gamma rays up to 11 MeV energy emerge that are detected during irradiation with a perpendicularly placed gamma detector, facilitating the qualitative and quantitative elemental composition determination of the irradiated volume. The elements are identified based on their gamma-ray energies using a spectroscopic library [31]. The elemental masses within the irradiated volume are derived from the net areas of the analytical gamma-ray peaks [32,33] and recomputed to atomic or mass fractions [34]. For method validation, we used an albite standard (Centre de Recherces Pétrographiques et Géochimiques AL-1) [35] and an ancient Chinese porcelain sample characterized in the IAEA CU-2206-06 Proficiency test [36].

NIPS-NORMA [37,38] of the Budapest Neutron Centre (BNC) is the only permanent facility designed for position-sensitive elemental composition measurements based on radiative neutron capture. This extension of the PGAA technique is called Prompt-gamma Activation Imaging (PGAI) [39]. For this purpose, in addition to the setup required for the PGAA element analysis of homogeneous samples (Compton-suppressed HPGe gamma-ray detector inside a massive lead shielding), the NIPS-NORMA facility is equipped with a large, 20 × 20 × 20 cm^3^ sample chamber, a xyzω motorized sample stage, a computer-controlled neutron slit to adjust the neutron spot size, and an optional neutron imaging camera placed downstream of the sample chamber. These hardware components are aligned with sub mm precision to the isocenter, that is, the geometric intersection of the neutron beam and the symmetry axis of the gamma detector’s collimator. This facilitates handling data from all modalities in a unified coordinate framework, directly correlating the motor positions, concentrations, and visual information without a registration process [40]. The sample positioning is based on either real-time visual feedback from the neutron imaging camera or employing a laser beam pointing along the centerline of the neutron beam (Figure 1b).

To maintain the firm placement and to avoid any damage to the upright standing sample during sample positioning, they were fixed to the sample manipulator using custom-made, disposable 3D-printed sample holders. Its upper part was developed using the exact complement of the artifact’s digital geometry model, while the bottom contains an interlock to mount it on the motorized sample stage [41]. Further, the measurement geometry, including the shape of the object taken from its scanned surface mesh, can also be reproduced digitally to allow the correction for both neutron- and gamma-ray-related matrix effects by MCNP6 [42] Monte Carlo computer simulations. This methodology is discussed in detail in our earlier publication [43].

The NIPS-NORMA station can be operated with thermal and cold neutrons, depending on the status of the cold source built into the core of the Budapest Research Reactor [44]. The energy distributions of the neutrons, and consequently, the penetration depths and the elemental sensitivities of these beams differ, even for the identical sample. Therefore, some PGAA measurements were made with thermal or cold neutrons, and in some cases, two measurements were completed at the same spot using the two different beam types. This is intended to verify that the PGAA method can generate bulk-representative results independently of the exact measurement conditions.

The irradiation spots of the PGAA measurements were set by considering the surface patterns on the porcelain, followed by a corresponding paint-free area as blank. The paint-free parts showed us the compositions of the bulk porcelains, while the difference between the two spots revealed the constituents of the paint. The neutron beam spot size was adjusted to provide the required discrimination and maximize productivity at the same time.

### 2.5. XRF Technique as a Surface-Analytical Tool

The XRF measurements were conducted in our lab with either an OLYMPUS Delta Premium or a Bruker Tracer 5g handheld X-ray fluorescence spectrometer [45]. In this case, as the surfaces of the decorated objects are not homogeneous, the information was carried by the spatial variation of the X-ray spectrum. Therefore, a handheld XRF device must be put close to the surface, held firmly, and positioned based on a video-feedback by the operator, or can be coupled to a computer-controlled, motorized xyz sample stage to precisely position the object relative to the sampling spot or make raster scanning element mapping [24,46,47] (Figure 1c). Although the X-ray spot size of such handheld XRF devices (3–8 mm) is larger than dedicated micro-XRF scanners [48], this class of equipment is still a viable, affordable, and portable solution for many heritage-science problems, especially if combined with sophisticated image post-processing [49].

**Figure 2:**
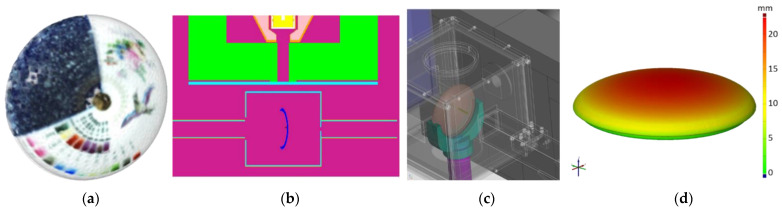
(**a**) the digital representation of the studied object obtained by 3D scanning, (**b**) the placement of the object in the MCNP6 simulation environment for PGAA matrix-effect correction, (**c**) the virtual model of the PGAI measurement geometry, visualizing the penetration of the pencil neutron beam, and (**d**) the elevation map for XRF scanning.

Our sample positioning device extends the macro-XRF (MA-XRF) approach to non-flat objects. An STL surface mesh from the 3D structured-light optical scan provides the elevation of the object as a function of (x,y)-coordinates in a given placement and relative to the sample stage’s baseplate (Figure 2d). This allows the adjustment of the z-axis of the sample stage to maintain the close contact of the spectrometer with the sample surface. In addition, we plan to add functionality to consider the local surface normal [26] so that this approach can facilitate MA-XRF scanning of convex objects.

The OLYMPUS Delta Premium device, equipped with a 40 kV X-ray source and a silicon drift detector, has internal calibration for Soil and Mining modes. The soil option was appropriate to determine elements K, Ca, P, Ti, Cr, Mn, Fe, Ni, Cu, and Pb, while the Mining mode, which uses a dual-energy beam, was applicable to metallic elements Ti, Cr, Mn, Fe, Co, Ni, Cu, and Pb. The Bruker Tracer 5g is a research-grade pXRF that has a 50 kV Rh X-ray tube and an extremely thin, 1 mm-tick graphene entry window. The latter has higher transmission throughout the X-ray’s energy spectrum and significantly improves the detection conditions for light elements. After acquiring the data, spectra were downloaded to the computer and evaluated by the bAxil software package [50].

## 3. Results and Discussion

**Figure 3:**
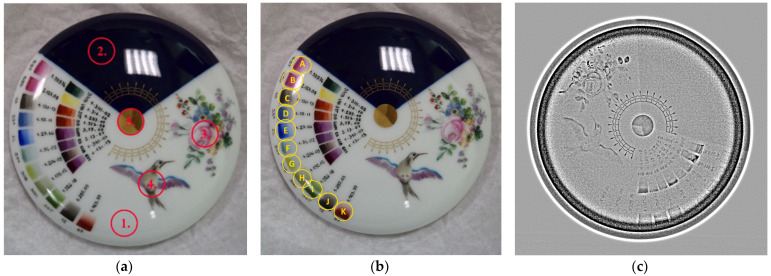
(**a**) PGAA measurement spots (1–5) selected for decoration, (**b**) color palette (A–K), and (**c**) dual-energy X-ray radiogram.

**Figure 4:**
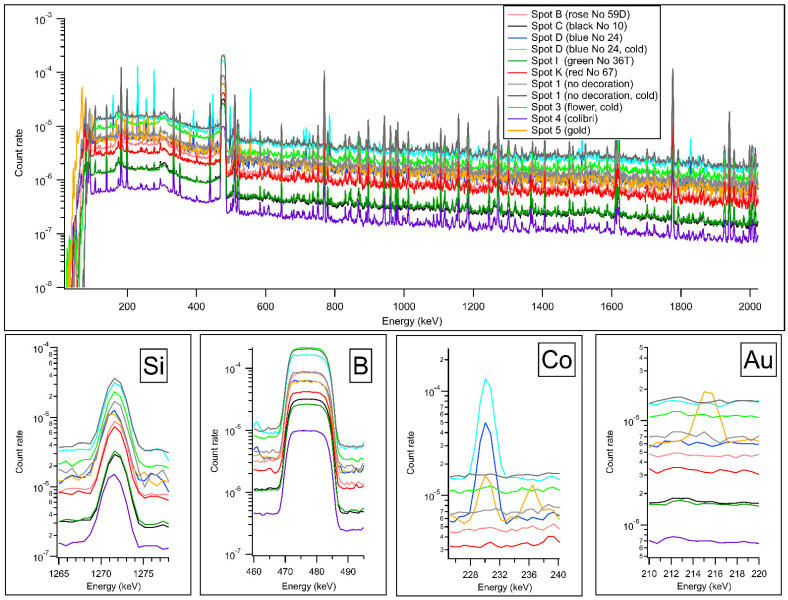
PGAA spectra up to 2 MeV, and the zoomed spectrum regions corresponding to bulk components Si, B, as well as elements Co and Au present in the surface decoration. PGAA data were taken with thermal neutron beam unless indicated otherwise in the legend.

The 3D scanned photorealistic digital model is provided in the Electronic Supplementary Materials. These scanned geometrical data served as input data to the measurement control and simulations, as described in Ref. [43] (Figure 2b,c). The measurement positions and dual-energy radiogram are visualized in Figure 3. Relevant parts of the PGAA spectra are plotted in Figure 4. After evaluation [34], we saw many common peaks attributed to matrix elements and a few new peaks appearing, characteristic of the elements present in the surface decoration. The quantitative results are listed in Table 1. The prompt-gamma concentration data for H, Na, Al, Si, K, Ca Ti, Fe, Nd, and Gd were found to be highly reproducible at Spots 1–5 of the artifact (as labeled in Figure 3a) and agreed within the error margin. A representative element of bulk is the Si peak in Figure 4.

The different neutron beam properties did not change the analysis results either. This confirms the robustness of the PGAA in carrying out bulk analyses even if other methods are influenced by the decorations. By comparing the paint-free and decorated areas, the presence of additional elements, i.e., Cu, Co, and Au, could be confirmed.

In the case of porcelains, the flux may typically contain a high mass fraction of B_2_O_3_: given the high sensitivity and ppm-level of the detection limit of the PGAA to element B, this is a unique technique to quantify this element. This feature is reflected in our results: the boron concentrations show a two-fold increase at positions 3 and 4, where the flower and bird motives are situated.

Regarding the palette area, labeled as Spots A to K in Figure 3b, we overlaid these PGAA spectra and the one corresponding to the paint-free areas, and they differed only in a few spectrum regions (Figure 4). This is, on the one hand, an easy and qualitative indicator of the inorganic components of the paint, but it also proves the ability of the PGAA technique to probe the subsurface composition well. In the case of organic pigments, the indicator element is Hydrogen, as it has much higher sensitivity than the corresponding O, C, and N elements. This means that the organic nature can be confirmed this way, but the exact classification of the organic paint requires the use of another technique, e.g., handheld Raman spectrometry. We positively identified Mn in the case of measurement spot 3 (gray); blue contained Co, while the red contained elevated concentrations of Fe compared to the bulk.

Although this sample contained < 1 ppm Cd, which is the typical DL of PGAA in this matrix, it was quantified with higher accuracy in many other traditional and contemporary porcelain items we recently analyzed in our lab.

**Figure 5:**
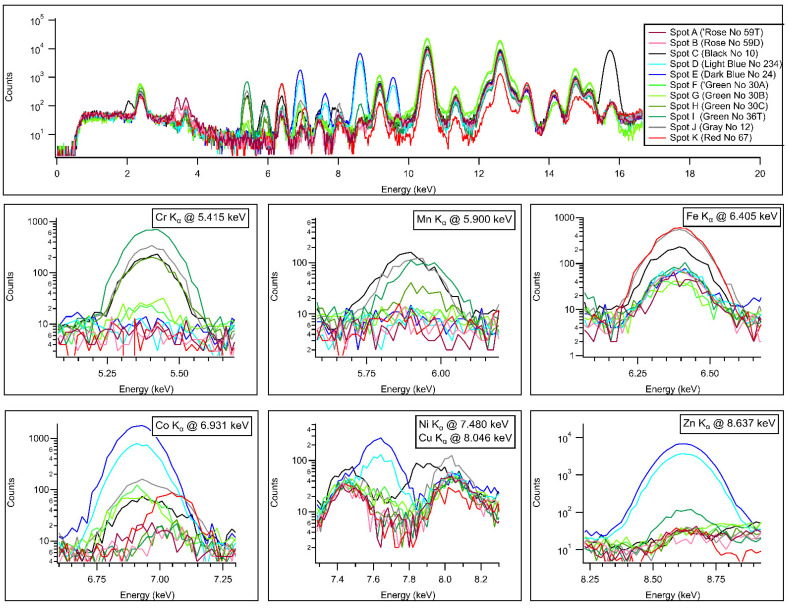
The K_α_ analytical lines of Cr, Mn, Fe, Co, Ni, Cu, and Zn in the 5–9 keV region of the XRF spectra for the color palette.

With XRF, whose spectra are plotted in Figure 5 and data listed in Table 2, there is a clear difference already between measurement points 0 (bare bulk without glaze) and 1 (white area covered with glaze) and an apparent negative bias of the major components Al. Si at decorated regions was observed, proving the presumption that the painted areas allowed the X-rays to penetrate less into the bulk. The overall scatter of the data was far larger than those of PGAA. However, due to the limited sampling depth, the XRF was more sensitive to indicate the surface variations and determined Sn that PGAA did not report. The detection limits for some elements were found to be better for XRF than for PGAA, but this also differs on the thickness of the bulk relative to the decorated layer. All green color shades contained Cr, highest in spot 8, while in the light green, one finds both Cr and Co. The gray paint contained Mn, Co, and Ni that PGAA was not able to detect. The dual-energy X-ray radiogram successfully revealed the thickness variation of the brushstrokes (Figure 3c).

**Figure 6:**
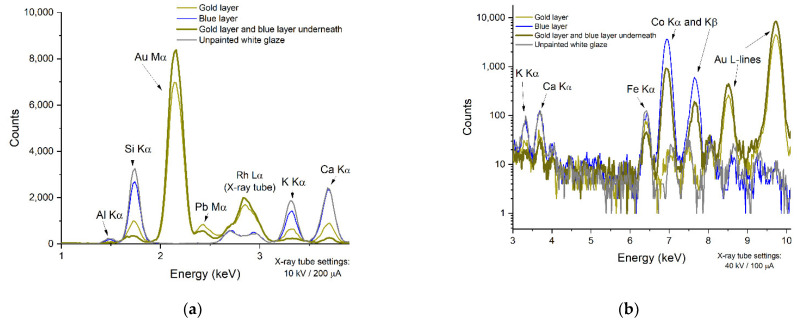
Two XRF spectra recorded at the 10 kV (**a**) and 40 kV (**b**) voltage settings at the central gilded area. Note the suppression of matrix peaks (Si, K, Ca) by the increasing thickness of golden/Sevres blue layers relative to the unpainted areas.

The PGAA spectrum taken at the central gilding indicated not only the presence of gold but also showed a strong correlation with the cobalt spectrum (see the correlated peaks at 230 and 236 keV of the golden-colored line in the Co plots of Figure 4), proving that underneath the gold layer the Sevres blue paint is present. This is also justified by the radiogram, as well as the anti-correlation of the matrix peaks Al, Si, K, and Ca with the thickness of the gilding/blue paint in the XRF spectrum (Figure 6). 

## 4. Conclusions

In addition to the main components of porcelain, such as Si, Al, K, Ca, Na, Fe, and Mg, PGAA could quantify multiple trace elements, mainly gadolinium, titanium, and in some pigments, cobalt, manganese, cadmium, and neodymium. In most of the cases, there were clear differences between the paint-free spots and decorated areas. Based on the PGAA results, we could differentiate between the decorated and non-decorated parts and proved that the blue paint contained cobalt, the pink and brown were manganese-based paints, the red paint contained Fe, the green paint used Cr, and the thin golden layers were also well detectable with Co-blue positively identified underneath.

The results of the pXRF measurements both supported and complemented the PGAA results. By choosing appropriate, glaze-free measurement points, we could achieve almost identical porcelain bulk compositions. This mostly remained true even if we measured the different surface patterns on the porcelains by PGAA. Most of the differences between the two techniques’ results could be explained by the differing probing volumes and the detectability conditions of the elements. Our data are compatible with the results of the detailed PIXE element mapping published by our French collaborators [26].

Overall, we can conclude that the PGAA and pXRF methods are complementary and help us to gain both surface and bulk-related information on the samples. In addition, the techniques can confirm the other one’s results, if not quantitatively, at least qualitatively, contributing to the comprehensive interpretation of the measurement results and fully non-destructive but detailed characterization of valuable artifacts.

## Figures and Tables

**Table 1:** Concentrations (m%) by PGAA at measurement spots shown in Figure 3a, together with their 1-sigma relative uncertainties.

	1				2				3				4		5	
Beam	Thermal		Cold		Thermal		Cold		Thermal		Cold		Thermal		Thermal	
	m%	unc%	m%	unc%	m%	unc%	m%	unc%	m%	unc%	m%	unc%	m%	unc%	m%	unc%
H	130 ppm	9	100 ppm	6	130 ppm	6	100 ppm	6	220 ppm	10.	210 ppm	6	100 ppm	5	260 ppm	7
B	60 ppm	0.9	63 ppm	0.8	59 ppm	0.8	58 ppm	1.0	121 ppm	0.9	106 ppm	0.9	110 ppm	0.9	62 ppm	1.0
Na	0.48	3.0	0.46	2.0	0.57	2.9	0.54	3.1	0.63	3.0	0.52	2.3	0.58	2.9	0.57	2.9
Mg							1.0	9.7					1.3	8.1		
Al	16.1	1.6	17.2	1.5	15.7	1.6	15.5	1.6	16	1.6	16	2.1	16	2.0	15.8	1.8
Si	29	1.6	28	1.6	29	1.6	29	1.7	29	1.7	29	1.9	28	1.7	29	1.7
K	2.4	1.9	2.4	1.9	2.4	2.0	2.3	1.9	2.4	1.9	2.5	2.0	2.4	2.3	2.5	3.1
Ca	2.5	3.0	2.7	2.5	2.6	3.1	2.5	2.6	2.6	2.9	2.6	2.8	2.4	2.6	2.6	3.6
Ti	110 ppm	16.9	120 ppm	9.0			120 ppm	10.6	80 ppm	22.0	140 ppm	11.7	120 ppm	10.4		
Mn											100 ppm	9	100 ppm	4.8		
Fe	0.24	9.3	0.24	4.1	0.20	12.1	0.25	6.5	0.23	8.0	0.21	7.3	0.22	4.9		
Cu									0.11	9.0						
Co					0.24	3.2	0.24	2.8			50 ppm	17	30 ppm	14.2	0.059	3.8
Nd			20 ppm	16.8							30 ppm	19				
Gd	3.9 ppm	6.2	4 ppm	9.4	3.8 ppm	6.9	3.4 ppm	7.3	4 ppm	13.0	3.7 ppm	6.2	1.8 ppm	7.0	4.1 ppm	5.2
Au															0.21	3.5

**Table 2:** Mass percentage (m%) concentrations by pXRF at measurement spots shown in Figure 3a, together with their 1-sigma relative uncertainties.

	0		1		2		3		4		5	
	m%	unc %	m%	unc %	m%	unc %	m%	unc %	m%	unc %	m%	unc %
Al	15.4	2.6	8.28	3.3	6.59	3.9	2.38	7.6	3.71	5.7	4.57	7.4
Si	29.5	1.0	42.2	0.7	35.9	0.8	22.6	0.9	20.1	1.0	4.6	2.4
K	1.74	1.7	3.61	1.1	2.36	1.3	1.97	1.0	0.616	3.2		
Ca	2.89	1.0	3.04	1.0	2.78	1.1	2.49	1.2	1.42	1.4		
Ti	0.089	14.7	0.0501	22.2	0.0711	14.9	0.0768	15.0	0.0534	18.4		
Mn							0.0116	30.2	0.0822	8.4		
Fe	0.312	2.9	0.229	3.1	0.156	3.8	0.12	4.2	0.409	2.2	0.365	7.1
Co					3.64	0.5			0.0652	5.1	6.81	1.2
Cu	0.0107	10.3	0.0104	10.6	0.0166	7.8	0.0171	7.0	0.0191	6.3		
Sn	0.0435	3.9	0.0319	4.7	0.0366	4.4	0.133	1.5	0.0382	4.2		
Au											40.5	0.5

## Supplementary Materials

The following supporting information can be downloaded at: https://www.mdpi.com/article/10.3390/ma15155106/s1, Figure S1: The digital geometry model of the investigated Sevres porcelain, obtained via 3D optical scanning.
